# Evaluation of the *Illumigene* Malaria LAMP: A Robust Molecular Diagnostic Tool for Malaria Parasites

**DOI:** 10.1038/srep36808

**Published:** 2016-11-09

**Authors:** Naomi W. Lucchi, Marie Gaye, Mammadou Alpha Diallo, Ira F. Goldman, Dragan Ljolje, Awa Bineta Deme, Aida Badiane, Yaye Die Ndiaye, John W. Barnwell, Venkatachalam Udhayakumar, Daouda Ndiaye

**Affiliations:** 1 Malaria Branch, Division of Parasitic Diseases and Malaria, Center for Global Health, Centers for Disease Control and Prevention, Atlanta, GA, United States; 2 Laboratory of Parasitology and Mycology, Université Cheikh Anta Diop (UCAD), Dakar, Senegal; 3 Atlanta Research and Education Foundation/VA Medical Center, Decatur GA, United States

## Abstract

Isothermal nucleic acid amplification assays such as the loop mediated isothermal amplification (LAMP), are well suited for field use as they do not require thermal cyclers to amplify the DNA. To further facilitate the use of LAMP assays in remote settings, simpler sample preparation methods and lyophilized reagents are required. The performance of a commercial malaria LAMP assay (***Illumi**gene* Malaria LAMP) was evaluated using two sample preparation workflows (simple filtration prep (SFP)) and gravity-driven filtration prep (GFP)) and pre-dispensed lyophilized reagents. Laboratory and clinical samples were tested in a field laboratory in Senegal and the results independently confirmed in a reference laboratory in the U.S.A. The ***Illumi**gene* Malaria LAMP assay was easily implemented in the clinical laboratory and gave similar results to a real-time PCR reference test with limits of detection of ≤2.0 parasites/μl depending on the sample preparation method used. This assay reliably detected *Plasmodium* sp. parasites in a simple low-tech format, providing a much needed alternative to the more complex molecular tests for malaria diagnosis.

Current malaria diagnostic tests rely on parasite detection by microscopy or antigen-based rapid diagnostic tests (RDT). Molecular methods such as polymerase chain reaction (PCR), mostly used in reference laboratory settings, have been shown to increase the sensitivity of detection. However, molecular tools are not commonly used in endemic countries as they require sophisticated laboratory capacity. The scope of performing molecular diagnosis in resource limited settings expanded when the loop mediated isothermal amplification (LAMP) technique was introduced to detect infectious disease agents^1^. Unlike PCR, which requires alternating temperature conditions, LAMP amplifies nucleic acids (RNA and DNA) at a constant temperature (isothermal), typically around 62 °C–65 °C. This facilitated further innovations to adapt this technology as a portable platform for field use^2^,^3^,^4^. The LAMP assay has been shown to be a highly sensitive and rapid molecular method and malaria LAMP assays have been developed for the detection of *Plasmodium* species^5^,^6^,^7^,^8^. In an effort to simplify the LAMP assay for use in resource limited countries, several different formats of LAMP have been investigated and evaluated, including the use of colorimetric high throughput assays such as hydroxynaphthol blue^9^ and the malachite green dyes^10^.

The field deployment of any molecular testing, including LAMP, should address certain challenges such as 1) simplification of sample preparation from blood to obtain amplifiable DNA, 2) assay reagent stability under ambient conditions, and 3) ease-of-use for the end-user. With the exception of the Loopamp MALARIA kit (Eiken Chemical Co.), which utilizes a simpler sample preparation method as well as lyophilized reagents, many of the malaria LAMP assays described to date still rely on the conventional DNA extraction methods and liquid-based assay reagents that require maintenance in the cold. The use of lyophilized reagents and simplified sample preparation methods has potential to make it easier to deploy the LAMP assays in resource limited regions.

Recently, another commercially available malaria LAMP kit, the ***Illumi**gene* Malaria LAMP, was developed by Meridian Bioscience Inc. (Cincinnati, OH). This kit consists of pre-dispensed, ambient temperature stable LAMP reagents designed to detect malaria parasites at the genus level. The LAMP assay is performed using the ***illumi**pro-10*™ Incubator/Reader, which is capable of testing 10 samples per run. The change in turbidity associated with LAMP assays, due to the magnesium-pyrophosphate build-up as a by-product, is measured by the ***illumi**pro-10*™ reader and a qualitative result is determined. Two simple centrifuge-free methods to extract DNA from EDTA whole blood were designed: a simple filtration method (***Illumi**gene* Malaria; herein referred to as SFP for simple filtration prep as shown in Fig. 1a) and a gravity-driven gel filtration column (***Illumi**gene* Malaria PLUS; herein referred to as GFP for gravity filtration prep, as shown in Fig. 1b). Both procedures rely on chemical lysis and produce amplifiable DNA within 10 minutes.

Here, we report results from the first evaluation, conducted in a clinical laboratory in Senegal, on the performance of the ***Illumi**gene* Malaria LAMP assay using both the SFP and GFP sample preparation methods.

## Results

### Cross-reactivity and Limits of Detection of the *Illumigene* Malaria LAMP

The cross-reactivity and limits of detection (LoD) experiments were performed by Meridian Biosciences with technical assistance from CDC prior to the field evaluation of the assays. Based on the *Plasmodium* target DNA sequence alignments, the LAMP targets selected for ***Illumi**gene* Malaria LAMP primers are highly conserved across the *Plasmodium* genus. No DNA sequence homology was observed with the selected targets other than the *Plasmodium* sequences using BLAST sequence analysis software at NCBI against the entire content of GenBank non-redundant sequences database. The assay was shown to detect all five human-infecting Plasmodium species: *P. falciparum, P. vivax, P. ovale, P. malariae* and *P. knowlesi*. No cross-reactivity was observed with human genomic DNA or with all the non-*Plasmodium* species tested. Using *P. falciparum* strain 3D7 DNA, the Limit of Detection (LoD) of the SFP and the GFP methods were determined to be 2.0 parasites/μl and 0.3 parasites/μl, respectively. LoD using the WHO standard were determined to be 4, 256 IU/μl (equivalent to 2 parasites/μl) for the SPF method and 851 IU/μl (0.4 parasites/μl) for the GFP method. The LoD using *P. vivax* DNA (India VII strain) was found to be 0.1 parasites/μL for both the SFP and GFP assays.

### Evaluation of the *Illumigene* Malaria LAMP Assays in a Clinical Laboratory in Senegal

A total of two hundred sixteen (216) whole blood and corresponding dried blood spots (DBS) samples were collected from 3 clinical sites in Senegal: Pikine, Rufisque and Thies. The three sites are found in a malaria hypo endemic area in which three human infecting species, *P. falciparum, P. malariae* and *P. ovale* are known to circulate. The parasite prevalence in Pikine and Rufisque is about 3% and it is about 1% in Thies. All the samples were tested in a clinical laboratory located in Thies, that routinely conducts microscopic and RDT evaluations. The whole blood was used for the LAMP assays while the DBS were utilized for the PET-PCR assay. Sixty six of the whole blood samples were utilized immediately upon collection or stored between 2–4 °C for1 to 7 days before use for ***Illumi**gene* LAMP (prospective samples). The remaining 150 blood samples were collected and evaluated by microscopy and then frozen at <20 °C until utilized for the LAMP assays which was within 30 days of storage (retrospective). The patient population included 84 females and 131 males (the gender of 1 patient was not defined). Only 209 samples were included in the analysis because 5 samples (3 positive, 2 negative by microscopy) were excluded because the incorrect assay program was used for GFP testing and 2 samples gave invalid results using the SFP method in that the assay control (run with every sample) gave invalid results. The SFP and GFP performance characteristics were compared to the local microscopy performed in the field. The collected DBS were send to the CDC to be evaluated using the PET-PCR assay. These assays were performed blinded to the microscopy and LAMP results obtained in the field.

### Performance Characteristics of the Illumigene Malaria LAMP by Specimen Storage

No significant difference was observed in the obtained specificity by using either prospective or retrospective samples with the GFP (p = 0.146) and SFP assays (p = 0.5921) compared to microscopy.

### Performance Characteristics of the Illumigene Malaria LAMP compared to microscopy and the PET-PCR

The species distribution of the 209 samples utilized in this study was 134 *P. falciparum*, 1 *P. ovale* and 1 mixed infection: *P. falciparum* and *P. malariae*. The average parasite density, as determined by microscopy, was 36,942 parasites/μl and range 40 to 404,000 parasites/μl. Both PET-PCR and the ***Illumi**gene* Malaria LAMP assays identified additional positive samples compared to microscopy; 11 by GFP method and 7 by both PET-PCR and SFP method. Table 1 and Fig. 2, summarizes the obtained sensitivity and specificity of ***Illumi**gene* Malaria LAMP and microscopy, compared to PET-PCR, used as the reference test.

### Discrepant samples

Twelve samples had discrepant results between the PET-PCR and LAMP tests, Table 2. Four PET-PCR samples of Ct values above 37.0 were shown to be negative by the LAMP assays while 8 samples negative by PET-PCR were shown to be positive by either the SFP (4/8) and/or the GFP assay (8/8).

### Comparison between the SFP and GFP Sample Preparation Methods

Both sample preparation methods were easy to perform in the field with minimum laboratory training. The GFP assay detected four more positive samples than the SFP assay (148 samples compared to 144). These four samples were also found to be negative by the PET-PCR assay, Table 2.

## Discussion

The ***Illumi**gene* Malaria LAMP assays are capable of detecting malaria infections at the genus level with analytical sensitivity (LoD) equal to (SFP assay) or below (GFP) the WHO recommended 2 parasites/μl^11^ while using two simple sample preparation procedures. Results were obtained in less than one hour, including the sample preparation steps. This report compared the performance of ***Illumi**gene* Malaria LAMP to the PET-PCR assay, a real-time PCR assay commonly used in research or reference labs. Not surprisingly, both SFP and GFP assays, like the reference test, PET-PCR assay, detected more positive samples than microscopy, the gold standard diagnostic test in many malaria endemic countries. The analytical sensitivity of the GFP assay was about 7 fold more than that of the simpler SFP assay, providing a much lower LoD of 0.3 parasites/μL than the SFP assay (2 parasites/μL). Indeed, this could explain the fact that the GFP assay detected 4 samples as positive that both the SFP and PET-PCR did not detect, as shown in Table 2. This was not surprising given that the GFP assay provides a cleaner DNA preparation due to the additional column purification step not found in the simpler ***Illumi**gene* Malaria method. Nonetheless, the parasite detection limits observed for the two sample preparation methods implies that these assays are capable of detecting low density infections below the detection limits of both microscopy and RDTs.

The PET-PCR assay detected as positive four samples that both the GFP and SFP did not identify as positive. This raises the question as to whether these were false positive samples by PET-PCR or if these were indeed positive samples that were missed by both the LAMP assays. The fact that these samples had high Ct (Table 2) indicate that, if these were indeed positive samples, they have very low parasite densities. These observations are not entirely surprising given that similar inconsistencies in PCR replicates of low parasitemia samples have been demonstrated^2^,^12^. The reproducibility of PCR assays in the detection of samples with very low parasitemia was shown to alternate between positive and negative in about 38% of PCR replicates tested^12^. Therefore, it is very possible that these were indeed positive samples of low parasitemia which if retested with the LAMP assays would probably give positive results however, we also cannot rule out false positivity by PET-PCR.

Sub-microscopic infections have been shown to occur in all transmission settings and the use of microscopy for epidemiological studies greatly underestimates the prevalence of malaria infections^13^. It is important that low density infections are detected, as they have been shown to contribute to as much as 20–50% of all human to mosquito transmission in very low transmission settings^14^. Several studies have demonstrated that molecular assays are more sensitive at detecting low density infections, often missed by microscopy^15^,^16^,^17^. The need to detect even asymptomatic low density infections in the wake of calls to eliminate malaria has led to the development of simple field-adaptable molecular assays for parasites detection. Our results demonstrate that the***Illumi**gene* Malaria LAMP assays are comparable in sensitivity and specificity to a real-time PCR assay (PET-PCR) which is more technically challenging. The LAMP technique, unlike many other molecular tests, is well suited for field laboratory use as it does not require a thermal cycler. Indeed, in this study, the LAMP assays were performed in a clinical laboratory in a field setting. Results from this evaluation clearly demonstrate that the ***Illumi**gene* Malaria LAMP assays are as sensitive as the reference test and therefore can be useful in the detection of asymptomatic cases that are often sub-microscopic. However, the described evaluation was not designed to address the utility of this test for detecting asymptomatic infection in field survey as the main objective of this first evaluation was to determine the performance of this test in a clinical laboratory setting in a malaria endemic country by comparing with microscopic diagnosis and a reference molecular test. Therefore, future studies aimed at evaluating these assays for detection of asymptomatic cases in both low and high transmission settings are warranted.

Several malaria LAMP assays have been described^2^,^3^,^6^,^7^,^9^,^18^,^19^,^20^,^21^,^22^,^23^,^24^. However, to date, the only truly field deployment commercial LAMP kits that we are aware of are the Loopamp MALARIA kit (Eiken Chemical Co)^2^,^25^,^26^ and the ***Illumi**gene* Malaria LAMP described here. Both of these LAMP platforms address the challenges associated with the field deployment of many molecular tests such as simplification of sample preparation from blood, reagent stability under ambient conditions, and ease-of-use for the end user, making it easier to deploy sensitive molecular assays in resource limited laboratory settings. By way of differences, the “PURE” sample preparation method utilized in the Loopamp MALARIA kit (Eiken Chemical Co) requires a 5-minutes heating step and the use of a series of interlocking tubes which is unlike the straightforward simpler methods utilized in the ***Illumi**gene* Malaria LAMP. Additionally, the amplification and readout steps in the ***Illumi**gene* Malaria LAMP are both performed in one platform resulting in an objective “positive” or “negative” readout similar to the use of a turbidimeter, in the Loopamp MALARIA kit (Eiken Chemical Co).

The current recommendations for case management of suspected malaria cases and passive case detection, even in low transmission settings, are still microscopy and quality-assured RDTs^11^. The objective of this test evaluation was not to change this recommendation for primary diagnosis but to demonstrate the feasibility and utility of simpler molecular assays such as LAMP assays for diagnosis of malaria in a point of care clinical laboratory setting. The results of this evaluation demonstrates that this test can improve the detection limit of the diagnostic test at point of care as they would allow for the detection of cases missed by microscopy and RDTs as demonstrated in our study. The increased sensitivity of this molecular test is certainly valuable for case management in non-endemic countries as most of the patients may be non-immune to malaria and this test can help to treat cases that will be missed by current standard primary diagnostic tests. However, the utility of this test for case management in an endemic setting will require further consideration from ministries of health and World Health Organization and their recommendations for appropriate use. Nevertheless, this new sensitive test provides opportunities for other use as the malaria control ecosystem includes several different operational scenarios which call for more sensitive detection tools. These include 1) epidemiological surveys in which a large proportion of sub-microscopic cases are missed, 2) elimination-certification process where finding the last parasite is necessary, in the detection of asymptomatic cases and in reactive case detection studies such as mass screen and treat (MSaT), 3) focal screen and treat (FSaT) studies in which individuals are screened with (FSaT) or without (MSaT) identification of an index case and the positive cases are treated^11^ and 4) in follow up tests post- treatment or in vaccine trials. All these scenarios require highly sensitive tests and the ***Illumi**gene* Malaria LAMP assays provides such a test due to its impressive limit of detection. However, like many other malaria LAMP platforms reported to date, the ***Illumi**gene* Malaria LAMP assays can test a limited number of samples per run. Therefore, scenarios that require the testing of a large number of samples may require other platforms such as the recently described colorimetric LAMP assays^9^,^10^ with the limitation that these high-throughput colorimetric malaria LAMP assays still require sample preparation and lyophilized reagents for ease of use in the field. As technology advances, it is possible to envision the possibility of adapting the ***Illumi**gene* reader to facilitate the testing of larger sample size.

Simpler sample collection methods such as the use of a finger prick blood and filter papers are preferable in field settings in resource limited regions. A limitation of this study is the fact that a venous blood collection was used, however, this does not mean that this is the recommended sample collection method for use with the ***Illumi**gene* Malaria LAMP assays and while we did not evaluate the use a finger prick sample in our study, we do not see any scientific reason why such a sample collection method would not work with the LAMP assays described here which require only 50 μL of blood.

The ***Illumi**gene* Malaria LAMP kit described here is designed to detect parasites of the *Plasmodium* genus and would serve as a screening test for malaria parasites infections. However, the development of species specific primers for species identification, is easily achievable. Several malaria LAMP assays have been described capable of detecting both the *Plasmodium* genus as well as the different human infecting species^3^,^5^,^6^,^7^,^8^,^27^. The need to identify the infecting species is important in situations where treatment is required post testing, in which case species identification is necessary in order to provide the correct antimalarial and in providing valuable information on the burden and relative distribution of malaria species. Nonetheless, a genus-specific assay has utility in studies that monitor and evaluate malaria control programs in which species identification might not be absolutely necessary. Furthermore, in a clinical setting, a simple, highly accurate, genus-specific LAMP assay has the ability to eliminate the need for significant expertise, training, and experience associated with microscopy, and can be an excellent safeguard against possible false-negative results associated with conventional tests optimized for detecting higher density symptomatic infections.

In conclusion, the ***Illumi**gene* Malaria LAMP assays can be used reliably for the detection of *Plasmodium* in a simple low-tech but sensitive assay format providing a much needed alternative to more complex molecular tests for malaria diagnosis. Additionally, introducing innovative diagnostics like ***Illumi**gene* Malaria LAMP eliminates challenges associated with conventional molecular tests and provides new opportunities to diagnose malaria in point of care settings in non-endemic and in endemic settings. Given the impressive low limits of detection and ease of use of these assays, it is reasonable to assume that these assays can provide sensitive options for the detection of asymptomatic cases in elimination settings; however, this requires appropriate field evaluations for such use.

## Methods

### Ethics

The study protocol was approved by the National Ethics Committee for Health Research, of the Republic of Senegal before participant recruitment and sample collection were initiated. CDC investigators did not participate in specimen collection or interact with study subjects and did not have access to personal identifying information. Therefore, their participation was determined to be non-engaged in human subjects’ research under CDC human subjects’ protections procedures. All participants, or their parents or guardians, provided written informed consent. All the methods were carried out in accordance with the approved guidelines.

### Study site, participants and sample collection

The study was conducted between September and December, 2015 in Hospital A. LeDantec Laboratoire de Parasitologie in Dakar, Senegal. Study participants were selected from health clinics in hypoendemic settings in Pikine, Rufisque and Thies, using the following criteria: patient age of ≥3 years, presence of fever at time of enrollment, or history of fever during the 48 previous hours, absence of severe illnesses and willingness to participate in the study, and consenting. The test plan was designed to evaluate the performance of both prospective and archived EDTA treated blood samples from symptomatic patients. Retrospective samples were collected previously, tested by microscopy and stored frozen (≤−20 °C) until testing with ***Illumi**gene* LAMP assays. For the prospective arm, a 5 mL volume of venous whole blood was collected in EDTA tubes and used to prepare both thick and thin blood smears for microscopy.

### *Illumigene* Malaria LAMP Assays

**Figure 1:**
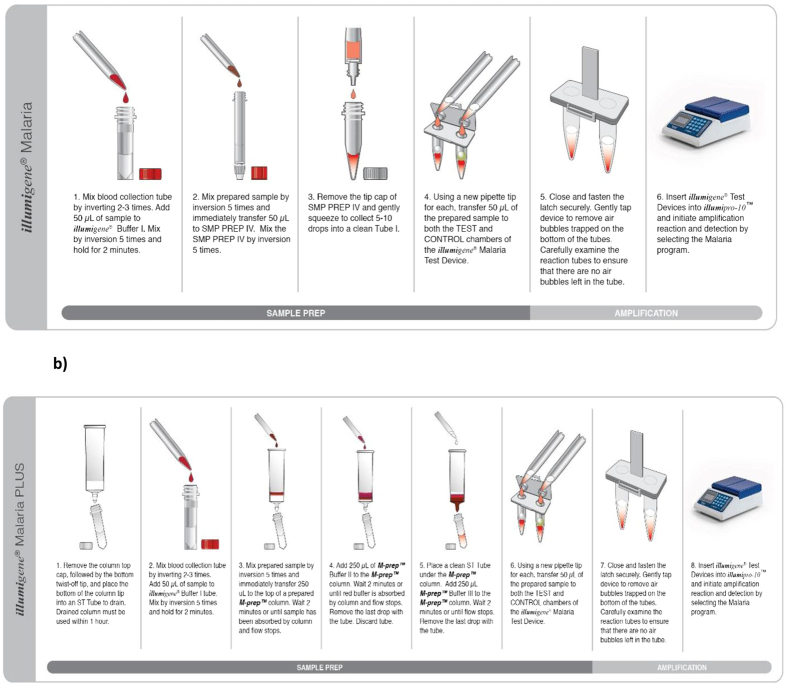
*Illumigene* Malaria LAMP workflow. The ***Illumi**gene* Malaria LAMP assay workflows include a sample preparation step followed by an amplification step. Two sample preparation workflows were developed: one using a simple filtration sample preparation method, Fig. 1a, and the other a 7–10 minutes gravity-driven gel filtration sample preparation method, Fig. 1b. The assays are performed as described in the corresponding insert. The necessary LAMP reagents are lyophilized in the ***Illumi**gene* Malaria Test Device which consist of a TEST tube with primers targeting the *Plasmodium* genus and a CONTROL tube with primers to detect the housekeeping human gene, used as a DNA isolation and amplification control. The runs are performed using the ***illumi**pro-10*™ incubator Reader and a qualitative result (positive, negative or invalid) is printed put after the run. These figures were prepared and provided by Meridian Bioscience, Inc.

**Figure 2:**
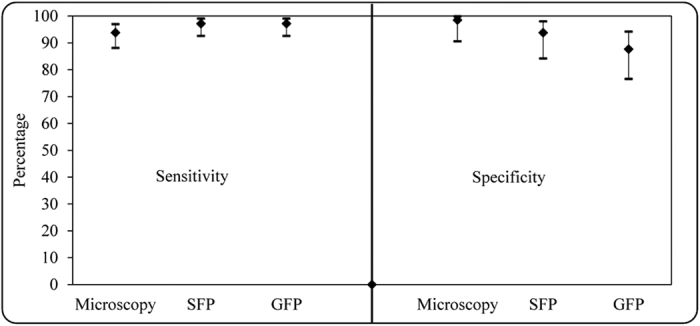
Graphical representation of sensitivity and specificity of microscopy, SFP and GFP compared to PET-PCR. The sensitivity and specificity of SFP (simple filtration prep), GFP (gravity-driven prep) methods and microscopy were calculated using the PET-PCR as the reference test. The whiskers show the 95% confidence intervals for sensitivity and specificity (centre diamonds).

The ***Illumi**gene* Malaria LAMP assay consist of two main steps: a sample preparation step and an amplification step, Fig. 1. Two centrifuge-free sample preparation methods were designed to extract DNA from whole blood collected in EDTA as anticoagulant: 1) a simple filtration method (***Illumi**gene* Malaria, herein referred to as SFP for simple filtration prep as shown in Fig. 1a) and a 7–10 minutes gravity-driven gel filtration method (***Illumi**gene* Malaria PLUS; herein referred to as GFP for gravity-driven filtration prep as shown in Fig. 1b). All the reagents and supplies are kept at room temperature. A blood sample is mixed with the provided lysis buffer and the lysate transferred to either SFP or GFP columns as described in Fig. 1. The collected eluates from each sample preparation were directly added to the ***Illumi**gene* Malaria test device consisting of lyophilized LAMP reagents. The lyophilized reagents in the LAMP device consist of the primers targeting the *Plasmodium* mitochondrial genome and the other necessary components including dNTPs and *Bst* DNA Polymerase to perform the LAMP assay. The control tube consist of LAMP reaction components and *Bst* Polymerase with primers to detect the housekeeping human gene, NADH dehydrogenase subunit 1. The design of this control supports that the whole blood extraction process step was performed correctly and identifies any potential sample inhibition.

The clinical trial site in Senegal utilized a full production lot of ***Illumi**gene* Malaria LAMP kits that were labeled for Investigational Use Only (IUO). The kits, the ***illumi**pro-10™* Incubator/Reader, and record sheets were shipped to the test site at the beginning the study. All tests were performed using the ***illumi**pro-10*™ incubator Reader, installed with Software version 2.00:602 and an IUO Malaria Assay protocol. External controls consisting of a known positive sample (Meridian Bioscience Inc. (Cincinnati, OH) and known non-malaria human blood were run on each day of patient testing. These samples were processed in the same manner as the patient samples. The runs were considered valid if the control test tube gave a positive result and if all the other controls gave the expected results. A qualitative result (positive, negative or invalid) was printed after every run.

### Microscopy

Thick and thin blood smears were prepared in the field for microscopy reading at the Laboratory of Parasitology and Mycology at Cheikh Anta Diop University (UCAD), A. Le Dantec Hospital in Dakar, Senegal. The blood smears were stained with 10% Giemsa for 10 minutes using WHO procedures http://www.who.int/malaria/publications/atoz/9241547820/en/. A thick blood smear was considered negative when the examination of 300 high power fields did not reveal asexual parasites or gametocytes. Parasite densities were calculated by counting the number of asexual parasites per 200 leukocytes (or per 500, if the count is less than 100 parasites or gametocytes per 200 leukocytes), assuming a leukocyte count of 8,000/μl. For quality control, all slides were read by two expert microscopists and a third microscopist settled any discrepant readings.

### Cross-reactivity and inclusivity studies

Cross reactivity studies were performed by Meridian during their primer/assay validation phase. This was tested using the five human-infecting *Plasmodium* spp. *P. falciparum, P. vivax, P. malariae, P. ovale* and *P. knowlesi* DNA (obtained from the CDC) and negative human whole blood specimens inoculated with non-*Plasmodium* organisms including bacterial or fungal organisms to a minimum concentration of 1.0 × 10^6^ CFU/mL, virus at a minimum of 1.0 × 10^5^ TCID_50_/mL, or protozoans to a minimum concentration of 1.0 × 10^5^ organisms/mL. Where whole organisms were not available, 1.0 × 10^6^ copies/mL for genomic DNA were tested. The following organisms were evaluated: *Babesia microti, Borrelia burgdorferi, Haemophilus influenza, Klebsiella pneumoniae, Leishmania donovani, Leishmania infantum, Leptospira interrogans, Mycobacterium smegmatis, Salmonella typhi, Staphylococcus aureus, Staphylococcus epidermidis, Streptococcus pneumoniae, Treponema pallidum, Trypanosoma cruzi, Trypanosoma rangeli, Vibrio parahaemolyticus*, Cytomegalovirus (CMV), Epstein-Barr virus (EBV), Hepatitis B virus, Hepatitis C virus, Herpes simplex virus 1 (HSV 1), HIV-1, Human Papilloma Virus (HPV), and Rubella virus. In addition, the following organisms were evaluated through in silico analysis since the organism or their DNA could not be obtained for testing: *Anaplasma phagocytophilium, Clostridium botulinum, Orientia tsutsugamushi, Rickettsia conorii, Rickettsia rickettsii, Rickettsia typhi*, Chikungunya virus, Dengue virus (types 1–4), West Nile virus, and Yellow Fever virus. Human genomic DNA was also tested at 1.0 × 10^6^ copies/mL.

### Determination of the Analytical Sensitivity

Limit of Detection (LoD) of the two sample preparation methods was also determined by Meridian during their primer/assay validation phase. Both *P. falciparum* (3D7 strain) and *P. vivax* (India VII strain) samples, obtained from the CDC, were utilized. At least twenty replicates were tested at different concentrations with both SFP and GFP methods. A minimum of 20 valid results per dilution were used to determine the LoD using a statistically-based methodology which allows for the determination of LoD with a 95% confidence interval. LoD was calculated using a linear logistic model that assesses the relationship between the probability of the response and the parasite concentration. In addition, a lyophilized *P. falciparum* WHO standard (National Institute for Biological Standards, Hertfordshire, England) was used to determine the LoD of the assays. The sample was reconstituted in sterile water and different dilutions, in International Units/mL, were made in uninfected human whole blood. Twenty replicates of appropriate dilutions were tested with SFP and GFP. LoD was defined as the lowest parasite concentration detected at a 95% confidence level.

### PET-PCR assay

Two dried blood spots were spotted with EDTA preserved blood from each of the clinical samples and stored at 4 °C. One of the dried blood spot was shipped to CDC, Atlanta for evaluation using the Plasmodium PET-PCR assay. DNA was extracted using the QIAamp blood kit (QIAGEN, Inc., Chatsworth, CA) according to the manufacturer’s instructions and stored at 4 °C until processed. Each sample was tested once. Briefly, the Plasmodium PET-PCR reaction was performed in a 20 μl reaction containing 2X TaqMan Environmental Master Mix 2.0 (Applied BioSystems), 250 nM each forward and reverse primer for *Plasmodium* genus and 2 μl of DNA template. The reactions were performed under the following cycling parameters: initial hot-start at 95 °C for 15 minutes, followed by 45 cycles of denaturation at 95 °C for 20 seconds, annealing at 60 °C for 40 seconds. Samples with a CT value of 40.0 or below were considered positive.

### Statistics

The sensitivity and specificity of ***Illumi**gene* Malaria LAMP assays was calculated using both microscopy and PET-PCR as reference tests. The percentage specificity and sensitivity were calculated using the formulae shown below:


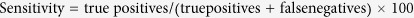



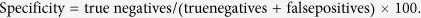


Probit analysis was used to determine the LoD for each ***Illumi**gene* Malaria LAMP assay. The LoDs were calculated using a linear logistic model that assesses the relationship between the probability of the response and the parasite density (parasites/μL). Data were entered into Microsoft Excel 2010 (Microsoft, Redmond, WA) and analysed using SPSS software, version 18 (SPSS Inc., Chicago, IL, USA). Precision of the estimates was determined by calculating exact 95% confidence intervals for each test statistic.

## Additional Information

**How to cite this article**: Lucchi, N. W. *et al*. Evaluation of the *illumigene* Malaria LAMP: A Robust Molecular Diagnostic Tool for Malaria Parasites. *Sci. Rep*. **6**, 36808; doi: 10.1038/srep36808 (2016).

**Publisher’s note:** Springer Nature remains neutral with regard to jurisdictional claims in published maps and institutional affiliations.

## Figures and Tables

**Table 1:** Performance of *illumigen*e Malaria LAMP assays and Microscopy compared to PET-PCR.

PET-PCR	GFP		
	Positive	Negative	Total
Positive	140	4	144
Negative	8	57	65
Total	148	61	209
			**95% CI**
Sensitivity	97.2%	140/144	92.6–99.1%
Specificity	87.7%	57/65	76.6–94.2%
	**SFP**		
Positive	140	4	144
Negative	4	61	65
Total	144	65	209
			**95% CI**
Sensitivity	97.2%	140/144	92.6–99.1%
Specificity	93.8%	61/65	84.2–98.0%
	**Microscopy**		
Positive	135	9	144
Negative	1	64	65
Total	136	73	209
			**95% CI**
Sensitivity	93.8%	135/144	88.1–97.0%
Specificity	98.5%	64/65	90.6–99.9%

**Table 2:** Discrepant results among the molecular assays (PET-PCR and LAMP assays).

Sample number	GFP	SFP	PET-PCR	PET-PCT Ct value
179	Positive	Positive	Negative	No Ct
193	Positive	Negative	Negative	No Ct
204	Negative	Negative	Positive	39.49
206	Negative	Negative	Positive	39.27
207	Negative	Negative	Positive	39.31
219	Positive	Negative	Negative	No Ct
256	Positive	Negative	Negative	No Ct
275	Positive	Positive	Negative	No Ct
276	Positive	Positive	Negative	No Ct
289	Positive	Negative	Negative	No Ct
430	Positive	Positive	Negative	No Ct
464	Negative	Negative	Positive	37.91
